# A Posteriori Dietary Patterns and Rheumatoid Arthritis Disease Activity: A Beneficial Role of Vegetable and Animal Unsaturated Fatty Acids

**DOI:** 10.3390/nu12123856

**Published:** 2020-12-17

**Authors:** Valeria Edefonti, Maria Parpinel, Monica Ferraroni, Patrizia Boracchi, Tommaso Schioppo, Isabella Scotti, Tania Ubiali, Walter Currenti, Orazio De Lucia, Maurizio Cutolo, Roberto Caporali, Francesca Ingegnoli

**Affiliations:** 1 Branch of Medical Statistics, Biometry, and Epidemiology “G. A. Maccacaro”, Department of Clinical Sciences and Community Health, Università degli Studi di Milano, Via A. Vanzetti 5, 20133 Milano, Italy; monica.ferraroni@unimi.it (M.F.); patrizia.boracchi@unimi.it (P.B.); 2 Department of Medicine, University of Udine, Via Colugna 50, 33100 Udine, Italy; maria.parpinel@uniud.it; 3 Division of Clinical Rheumatology, ASST Pini-CTO, Via G. Pini 9, 20122 Milano, Italy; tommaso.schioppo@unimi.it (T.S.); isascotti@hotmail.com (I.S.); tania.ubiali@gmail.com (T.U.); orazio.delucia@asst-pini-cto.it (O.D.L.); roberto.caporali@unimi.it (R.C.); francesca.ingegnoli@unimi.it (F.I.); 4 Research Center for Adult and Pediatric Rheumatic Diseases and Research Center for Environmental Health Department of Clinical Sciences and Community Health, Università degli Studi di Milano, Via G. Pini 9, 20122 Milano, Italy; 5 Department of Biomedical and Biotechnological Sciences, University of Catania, Via S. Sofia 97, 95123 Catania, Italy; walter.currenti@studium.unict.it; 6 Research Laboratories and Academic Division of Rheumatology, Department of Internal Medicine, University of Genova—IRCCS San Martino Polyclinic Hospital, Viale Benedetto XV 6, 16132 Genova, Italy; mcutolo@unige.it

**Keywords:** cross-sectional study, a posteriori dietary patterns, diet, disease activity, DAS28, SDAI, factor analysis, rheumatoid arthritis

## Abstract

To our knowledge, no studies have investigated the relationship between a posteriori dietary patterns (DPs)—representing current dietary behavior—and disease activity in patients with rheumatoid arthritis (RA). We analyzed data from a recent Italian cross-sectional study including 365 RA patients (median age: 58.46 years, 78.63% females). Prevalent DPs were identified through principal component factor analysis on 33 nutrients. RA activity was measured according to the Disease Activity Score on 28 joints (DAS28) and the Simplified Disease Activity Index (SDAI). Single DPs were related to disease activity through linear and logistic regression models, adjusted for the remaining DPs and confounders. We identified five DPs (~80% variance explained). Among them, Vegetable unsaturated fatty acids (VUFA) and Animal unsaturated fatty acids (AUFA) DPs were inversely related to DAS28 in the overall analysis, and in the more severe or long-standing RA subgroups; the highest score reductions (VUFA: 0.81, AUFA: 0.71) were reached for the long-standing RA. The SDAI was inversely related with these DPs in subgroups only. This Italian study shows that scoring high on DPs based on unsaturated fats from either source provides independent beneficial effects of clinical relevance on RA disease activity, thus strengthening evidence on the topic.

## 1. Introduction

Rheumatoid arthritis (RA) is a chronic immune-mediated disease, primarily characterized by synovial inflammation, which leads to a progressive joint damage; the disease activity impairs physical function, activities of daily living, and quality of life [1,2].

Despite the increasing therapeutic armamentarium, a significant proportion of RA patients have an inadequate response to the available disease modifying anti-rheumatic drugs (DMARDs) [2]. In an attempt to relieve their symptoms, these patients make enquires to their rheumatologists about dietary modifications that might help them with symptoms. A recent survey capturing RA patients’ perspectives suggests nutritional advice being among relevant topics for future research [3]. Websites and leaf letters from patients’ associations often suggest foods, nutrients, or dietary patterns (DPs) to relieve symptoms [4,5]. The increasing recognition of this unmet need and of “unorthodox” dietary “therapies”—as adjuvant or replacement to traditional treatments—have recently led the scientific community to strengthen the evidence supporting dietary intakes as potential complementary therapies for RA [6,7,8,9,10,11].

Since early 1990 [12], most of the efforts were devoted to transpose results from in vitro or animal studies in short-term dietary interventions for RA activity control. Common dietary programs targeted either single dietary components—such as omega-3 fatty acids, anti-oxidant vitamins, or minerals—or DPs, including variants of vegetarian (lacto-ovo-vegetarian, vegan with or without gluten, strictly vegan, also combined with fasting), Mediterranean-type, and elemental eating plans, fasting and elimination diets [6,7,8,9,13,14]. Notably, the translation of DP-based interventions into clinical practice is extremely challenging [9]. Several factors related to previous clinical trials have hampered this issue, including the heterogeneity of clinical phenotypes (e.g., geographic region, disease duration, and severity), outcome measures, and dietary interventions (e.g., length, type, or doses), or flaws in trial design (e.g., small sample size or single center) and/or implementation (e.g., differences in major covariates after randomization, possible confounding effects during the intervention period, or high drop-out rates) [7,13].

With the exception of one cross-sectional study [15], the few available observational studies on RA mainly focused on disease onset [6,7,8,9]. However, from both a patient-centric and a public health perspective, even a minor real-life dietary effect, which is persistent and spanning over the disease course, is likely to be of great importance and to improve long-term physical function, quality of life, and other objective and subjective outcome measures [16].

Over the last twenty years, the analysis of DPs—combinations of dietary components intended to represent the total diet or key aspect of the diet—has provided a valuable complementary strategy to the traditional single-nutrient or single-food approach in chronic disease prevention [17]. Indeed, free-living people do not eat isolated nutrients or foods, but they eat meals consisting of a variety of foods with complex combinations of nutrients that are likely to be interactive or synergistic. Use of DPs captures the intrinsic complexity of diet and the potential synergistic effects between its different components [18]. In addition, results from epidemiological studies on the association between DPs and diseases can be more easily translated into dietary practice [19].

A few previous attempts have assessed the role of DPs in RA activity [16,20,21,22,23,24,25], or development [26,27,28]. Studies on RA disease activity have considered the effect of adherence to a Mediterranean [16,20,21,25] or a vegetarian/vegan [22,23,24] DP. In either case, individual diets are compared against [20,25] or modified to comply with (experimental group) [16,21,22,23,24] evidence-based benchmark diets, within the so-called a priori DP approach.

To our knowledge, no study so far has derived a posteriori DPs and assessed their relationship with RA development or activity. The a posteriori DPs are obtained from the application of multivariate statistics (e.g., principal component analysis, exploratory factor analysis, or cluster analysis) to the available dietary data [29]. Therefore, the a posteriori DPs synthesize the different aspects of the actual dietary behavior, as measured at any single time-point reflecting recent dietary habits of a population. In detail, we will represent the actual diet of a large sample of RA patients from northern Italy, where lifelong adherence to Mediterranean-style DPs is more likely to be observed and to contribute to the expression and severity of RA [30].

In addition, when factor analysis is applied to derive a posteriori DPs, subject’s overall diet is disentangled into a set of different and independent DPs, each capturing specific dietary behaviors. As compared to any a priori DP, this approach is promising because it allows to identify prevalent DPs in a population, to separate out specific aspects of actual dietary behavior into separate DPs, and to relate each DP to RA activity, after a stronger control for all the other DPs identified in the population.

In the context of the current evidence [31], we will provide an overall picture of DPs identified in this large sample of RA patients from northern Italy, and we will assess the effect of each actual DP on RA activity, after controlling for the remaining DPs, as well medication, disease history, lifestyle habits, and anthropometric data.

Although existing data suggest a modest effect of diet at best [6], as confirmed also in previous evidence on a priori DPs measuring objective measures [20,25], breaking through this analysis plan will confirm or improve our understanding on which DPs should be followed for long-term management of diet in RA patients.

## 2. Materials and Methods

### 2.1. Design and Participants

This was an observational, cross-sectional, single-center study approved by the local ethical committee (granted ethical approval: 751_2017, Comitato Etico Milano Area 2). The study population consisted of all consecutive patients aged between 18 and 65 years with disease duration ≥ 3 months who met the 1987 American College of Rheumatology (ACR) [32] and/or the 2010 ACR/European League Against Rheumatism classification criteria [33] for RA and referred to our in- and out-patient rheumatology clinic at Gaetano Pini Hospital in Milan, Italy, from January 2018 to December 2019. Details on selected characteristics of the study participants are provided in Table S1.

The results on a subset of subjects included the current study (205 RA patients, median age: 53 years, 80.49% females) were recently published in a paper [20] that assessed the effect of the adherence to the Mediterranean diet and RA perception/disease activity.

More generally, data were collected within a broader study which includes an ongoing follow-up with clinical and laboratory evaluations.

### 2.2. Data Collection

Participants’ data were initially collected and later updated during routine doctor visits by centrally trained personnel through a structured interview assessing information on sociodemographic characteristics, anthropometric factors, cigarette smoking and alcohol drinking, and a personal medical history of selected comorbidities and other potentially relevant diseases. In addition, RA features, RA disease activity (Disease Activity Score in 28 joints (DAS28) with C-reactive protein (CRP) and Simplified Disease Activity Index (SDAI)), on-going RA treatment, patient’s general health (0 (worst imaginable health state)—100 (best imaginable health state)), and physician’s global assessment (0 (best disease control)—10 (worst disease control)) were carefully assessed at each visit, together with relevant laboratory parameters (including CRP and Erythrocyte Sedimentation Rate (ESR)).

Dietary habits in the previous six months were also collected with a 110-item food frequency questionnaire (FFQ), whose reproducibility and relative validity were assessed in an Italian population from the Sicily region, with good results [34]. The FFQ included the following sections: (I) meat and fish products; (II) sweets, nuts, and snacks; (III) oils and seasonings; (IV) vegetables; (V) fruit; (VI) drinks; (VII) cereals and starchy foods; (VIII) milk and dairy products. Participants were asked how often, on average, they had consumed the foods and beverages included in the FFQ, with nine responses ranging from “never” to “4–5 times per day”. A medium serving size in grams (e.g., tomatoes, 100 g) or in natural units (e.g., coffee, one cup) was provided for each FFQ item. Consumption of foods in season was indicated within the FFQ, to account for the food supply; corresponding estimates of the average food consumption were adjusted accordingly [34]. Participants filled in the FFQ, while they were waiting for the routine examination, and, in case of any doubt, they were referred to the doctor in charge of their visit.

For each participant, we estimated total energy and selected nutrient intakes after transforming the daily frequency of consumption of each FFQ item through the food composition tables [35] produced by the Italian Research Center for Foods and Nutrition [36] and the US Department of Agriculture (USDA) National Nutrient Database for Standard Reference, version 2011 [37], when needed. Content in foods of flavonoids, phenolic acids, and tyrosols were retrieved from the Phenol-Explorer database [38] (see [39] for additional details on habitual flavonoid intake estimates).

When incomplete or unreliable FFQs were identified within the subset of 39 subjects showing extreme (<5th or >95th percentile) total energy intakes at baseline, corresponding subjects (1 subject only, with total energy intake equal to 762.63 kcal) were excluded from the analysis; this leaves a total of 365 subjects who contributed to the baseline dataset.

### 2.3. Statistical Analysis

#### 2.3.1. Factorability of the Original Matrix

The analysis was carried out on a comprehensive list of 33 macro-nutrients, micro-nutrients, minerals, and other food compounds (for simplicity, we referred to this composite set of dietary components as “nutrients” hereafter). We examined the potential relationships among nutrients to avoid over-representing specific profiles of consumption, thus resulting in artificially higher correlation coefficients; we also targeted specific nutrients or profiles of consumption (e.g., fatty acids), known to be involved in RA disease activity or development from the literature (e.g., [6,7,8,9,10,11]). We evaluated the factorability of the nutrient-based correlation matrix by visual inspection and through statistical procedures, namely Bartlett’s test of sphericity, overall (or Kaiser-Meyer-Olkin) and individual measures of sampling adequacy [40]. Given the satisfactory results that we obtained (see Table S2), we applied an exploratory principal component factor analysis to derive the a posteriori DPs on the nutrient-based correlation matrix.

#### 2.3.2. Dietary Pattern Identification: Principal Component Factor Analysis

The correlation structure of the 33 selected nutrients was described in terms of a smaller number of underlying unobservable and randomly varying factors [41], which can be interpreted as DPs derived from factor analysis.

The principal component method was used for DP identification in the main factor analysis. We selected the number of factors to retain taking into account the following criteria: factor eigenvalue >1, scree-plot visual inspection, and factors interpretability [41]. We applied a varimax rotation to obtain a simpler loading structure, likely providing a better interpretation of factors. Factor labeling was based on nutrients with rotated factor loadings ≥0.63 in absolute value. We set this cut-off because it implies a minimum contribution of any factor to any nutrient’s total variance of approximately 40% (i.e., 0.63^2^) [42]. Nutrients with factor loadings ≥0.63 (absolute value) are called ‘dominant nutrients’ hereafter.

Factor scores were estimated for each participant and each DP. They quantify the degree of adherence of each subject’s diet to each identified DP. Factor scores were computed using the weighted least squares method and, by design, they are continuous measures with mean equal to 0 [43,44,45].

#### 2.3.3. Reproducibility, Reliability, and Validity of Dietary Patterns

To evaluate the internal reproducibility of the identified DPs [43,44,45], we performed additional analyses using: 1. different estimation methods (namely principal axis factor analysis with generalized least squares estimation method, and maximum likelihood factor analysis, after logarithmic transformation of the original nutrients to improve adherence to the Normality assumption), and 2. a different procedure for estimating factor scores (namely multiple regression method) [41]. We also carried out two separate analyses on females and males.

Since all these checks were satisfactory and results were similar in the male and female subsamples, we performed all the subsequent analyses on factor scores obtained from the main analysis, based on a principal component factor analysis on all the subjects available at baseline, with varimax rotation and weighted least squares method.

To evaluate factor reliability and refine the identified DPs [43,44,45], we calculated the standardized Cronbach’s coefficient alpha for those nutrients with a factor loading ≥ 0.40 in absolute value on any factor [46]. For each factor we computed the overall alpha coefficient and all available coefficient alphas when-item-deleted, which assessed the importance of each nutrient within the corresponding DP.

To further interpret the identified DPs [43,44,45], we calculated the Spearman rank correlation coefficients between the continuous factor scores derived from principal component factor analysis and the daily intake of 33 selected food groups and condiments, defined on the same data and derived from the original 110 food items.

#### 2.3.4. Risk Estimates

For each DP, participants were categorized into three groups based on the tertiles of the distribution of each factor score. The highest categories of consumption (vs. the reference category representing the lowest consumption) were entered as independent variables into multiple regression models that assessed the effect of adhering to each DP on RA disease activity—the dependent variable here measured by either DAS28-CRP or SDAI.

When RA disease activity was entered in continuous in the regression models, we estimated the effect of the highest consumption categories on a 1-point increment/decrease in RA disease activity. Violations of the standard ordinary least squares assumptions suggested us to use the robust MM estimator for our data [47]. When RA disease activity was modeled as a discrete (dichotomous) variable (e.g., presence of low, moderate, or high disease activity vs. remission), we estimated the odds ratios (ORs) of RA disease activity (vs. remission) and the corresponding 95% confidence intervals (CIs) for the highest categories of each factor score (vs. the lowest one) within unconditional multiple logistic regression models. In the current analysis, disease activity—as measured with DAS28-CRP and SDAI—was dichotomized because combining subjects on moderate or high disease activity had provided only 21 and 28% of the total frequency for each outcome, as compared to 62 and 30% of subjects in remission, respectively (see Table S1 for details).

We fitted both separate models for each factor and a composite model including all the factors simultaneously. We included in each model the following potential confounding variables: age (≤55, >55 years old), sex, education (maximum level attained: primary school, middle school, high school, university), body mass index (BMI, <18.5, 18.5–24, 25–29, ≥30 kg/m^2^), cigarette smoking status (never, former, current), alcohol drinking intensity (never drinker, <1, 1–<2, ≥2 drinks/day, where 1 drink/day was equal to 12 g of ethanol in the Italian population [48]), disease duration (≤5, 5–≤10, 10–≤15, 15–≤25, >25 years), rheumatoid factor (RF) (negative, positive), anti-citrullinated protein antibodies (ACPA) (negative, positive), presence of any therapy (yes, no), conventional synthetic (cs)DMARDs (no, yes), biologic (b)DMARDs (no, yes), targeted synthetic (ts)DMARDs (no, yes), steroids (no, yes).

Stratified analyses were carried out by RF and/or ACPA values (RF and ACPA negative, RF and/or ACPA positive) and by disease duration (≤15, >15 years); heterogeneity across strata was tested with the likelihood ratio test. Two sensitivity analyses were also conducted on participants who had normal blood pressure or did not report either gastro-esophageal reflux or gastritis.

Calculations were performed using the open-source statistical computing environment R [49], with its libraries psych [50] and GPArotation [51].

## 3. Results

Table S1 shows descriptive statistics and/or frequency distributions of several characteristics of the Italian RA patients included in the current study. Briefly, the median age at baseline visit was 58.46 (Interquartile range (IQR): 47.81−69.03 years), females were 78.63%, and most of the participants (60.55%) finished the high school and/or the university. The median BMI was 23.63 (IQR: 21.00−26.78) Kg/m^2^; never smokers or drinkers were 51.64% and 29.04%, respectively. The median disease duration was 12.81 (IQR: 8.08−20.72) years, with RF positivity being 53.70% and ACPA positivity 50.96%. Disease activity measures DAS28-CRP and SDAI showed medians of 2.21 (IQR: 1.61−3.02) and 6.30 (IQR: 3.01−11.81), with high disease activity present in 3.84% and 5.75% of the sample, respectively. Major comorbidities included arterial hypertension (33.42%), gastro-esophageal reflux (19.18%), and gastritis (8.77%).

Visual inspection and results from statistical procedures for matrix factorability (Table S2) suggested that the nutrient-based correlation matrix was adequate to carry out a factor analysis. In detail, the Bartlett’s test of sphericity was statistically significant (*p*-value < 0.001), allowing rejecting the null hypothesis that the correlation matrix is the identity matrix. The overall measure of sampling adequacy was equal to 0.88, thus indicating that the sample size was appropriate, as compared to the number of nutrients included in the current analysis. Moreover, the individual measures of sampling adequacy were generally satisfactory: 13 nutrients had measures ≥0.90, 15 between 0.80 and 0.89, 4 had measures between 0.70 and 0.79, and only phenolic acids had a measure below 0.60.

Table 1 gives the factor loading matrix of the five retained DPs, and the corresponding communalities. The identified DPs accounted for ~80% of the variance of the original nutrients. Any examined nutrient had one or more factor loadings ≥0.30, thus suggesting it captured a relevant aspect of the overall diet. The greater (in absolute value) was the loading of a given nutrient to a factor, the higher was the contribution of this nutrient to the factor. The first DP was labeled as “Anti-oxidant vitamins and fiber”, as it was characterized by high (negative) loadings on soluble carbohydrates, potassium, vitamin C, vitamin A—Retinol Activity Equivalent, soluble and insoluble fiber, lignans, and flavonoids. The second DP, named “Starch-rich”, was characterized by high (positive) factor loadings on total protein, starch, sodium, phosphorus, iron, zinc, magnesium, selenium, thiamin (vitamin B1), and niacin. The third DP, “Vegetable unsaturated fatty acids” (VUFA), had high (negative) factor loadings on linoleic and linolenic fatty acids and vitamin E, whereas the fourth DP, “Animal unsaturated fatty acids” (AUFA), showed high positive factor loadings on eicosapentaenoic acid (EPA), docosahexaenoic acid (DHA), and vitamin D. Finally, the fifth DP, named “Animal products”, had high (negative) loadings on cholesterol and saturated fatty acids.

Standardized Cronbach’s coefficient alphas ranged from 0.89 to 0.98 across the five identified DPs; most standardized coefficient alphas when-item-deleted were smaller than the corresponding coefficient alpha for the same DP. Both results suggested a satisfactory internal consistency of the identified DPs (data not shown).

Table 2 presents the Spearman rank correlation coefficients between the continuous factor scores, as identified from the nutrient-based principal component factor analysis, and a set of 33 selected food groups and dressings measured on the same subjects with the same FFQ. The “Anti-oxidant vitamins and fiber” DP score was positively correlated with consumption of fruit available in winter (i.e., citrus fruit), summer (e.g., peaches, apricot, and melon) and all seasons (i.e., apples, pears, bananas, and kiwi fruit), of other vegetables, leafy vegetables, cabbages, and legumes. The “Starch-rich” DP score had positive correlation coefficients with refined grains, whole grains (from bread, rice, and pasta, as well as pizza and breakfast cereals), and potatoes. The VUFA DP showed positive correlations with nuts and the olive oil and olives food group, whereas the AUFA DP with fish in general, with very high values for fatty and lean fish, and seafood close to 0.30 too. The “Animal products” DP was positively correlated with the consumption of hard and soft cheese, red meat, milk and yogurt, sweets, and the butter and margarine food group.

Table 3 and Table 4 show the ORs (and the corresponding 95% CIs) of RA disease activity (upper panel) and the increment in the mean outcome disease activity scores, DAS-CRP and SDAI in continuous (lower panel), according to the highest tertile-based categories of consumption of the five retained DPs. The results refer to the composite models including all the DPs simultaneously, and all the mentioned confounding factors. The results concern the overall sample (left part of the table) and the stratified analyses on the more severe (center) and long-standing (right part of the table) RA variants. The results from the stratified analyses on RF and ACPA negative subjects and shorter (i.e., ≤15 years) disease durations were presented in Table S3 for DAS28-CRP and in Table S4 for SDAI.

Table 3 shows a consistent and protective effect of an increasing consumption of the VUFA DP on DAS-CRP: in the main analysis (left part of the table), the OR of disease activity was equal to 0.39 (95% CI: 0.21–0.74, for the highest versus the lowest consumption category) and the beta coefficient was equal to −0.36 [Standard Error (SE): 0.14], with a *p*-value from Student’s *t*-test < 0.001. In addition, scoring high on the AUFA DP provided a protective effect on DAS-CRP, with an OR of borderline significance (0.53, 95% CI: 0.28–1.00) and a significant beta coefficient equal to −0.25 (SE: 0.14), *p*-value < 0.05. No significant heterogeneity was found across strata of RA severity or duration in either logistic or linear regression models (all *p*-values of heterogeneity across strata >0.10 for any of the identified DPs). However, results for the VUFA and AUFA DPs appeared stronger in more severe (center) or long-standing (right part of the table) RA forms. In detail, when DAS-CRP was measured on participants with longer disease durations, in logistic regression models, the risks halved those of the main analysis (AUFA DP: OR = 0.29, 95% CI: 0.09–0.92), or represented an even stronger reduction (VUFA DP: OR = 0.10, 95% CI: 0.03–0.36, for the highest consumption category of both DPs); similarly, in robust regression, the beta coefficients doubled (in absolute value), being equal to −0.81 (SE: 0.25, *p* < 0.001) for the VUFA and −0.71 (SE: 0.24, *p* < 0.001) for the AUFA DP, respectively. The results were, however, based on smaller sample sizes, as compared to the main analysis. The remaining DPs—“Anti-oxidant vitamins and fiber”, “Starch-rich”, and “Animal products”—were materially unrelated with RA disease activity, except for a detrimental effect (beta = 0.37, SE: 0.20, *p*-value < 0.05) for the “Anti-oxidant vitamins and fiber” DP in the more severe form of RA.

Table 4 shows the ORs of disease activity and the beta coefficients obtained from the regression models including the SDAI as dependent variable. A higher consumption of the dominant nutrients for the VUFA and AUFA DPs was still associated with a lower risk of SDAI-based disease activity, but the effect was more pronounced in the stratified analyses: the RF and/or ACPA positive subjects showed an OR equal to 0.36 (95% CI: 0.14–0.93) for the VUFA DP and subjects with longer disease durations showed an OR of borderline significance for the AUFA DP (OR = 0.27, 95% CI: 0.07–1.02), in the absence of heterogeneity across strata (all *p*-values > 0.10 for the identified DPs). The results from the robust linear regression models were in the same direction, but the stratified analyses showed even stronger decreases in disease activity, confirmed by heterogeneity detected across strata of both RA severity and duration. The highest categories of consumption of VUFA and AUFA DPs reached beta coefficients equal to −2.78 (SE: 1.22, *p*-value < 0.01) and −3.85 (SE: 1.27, *p*-value < 0.001) in RF and/or ACPA positive subjects and equal to −4.60 (SE: 1.66, *p*-value < 0.001) and −4.75 (SE: 1.61, *p*-value < 0.001) for longer durations, respectively.

In addition, participants showing an intermediate score on the “Animal products” DP were likely protected against development of (SDAI-based) disease activity in the main analysis (OR = 0.46, 95% CI: 0.23–0.94 and beta = −1.51, SE: 0.83, *p*-value < 0.05), in RF and ACPA negative subjects (OR=0.21, 95% CI: 0.05–0.85 and beta = −2.07, SE: 1.43, nonsignificant *p*-value, see Table S4), and for longer disease durations (OR= 0.22, 95% CI: 0.05–1.00 and beta = −3.01, SE: 1.63, *p*-value < 0.05). Finally, the “Starch-rich” and the “Anti-oxidant vitamins and fiber” DPs were materially unrelated with RA disease activity; however, in linear regression models only, intermediate categories of consumption of these DPs showed a protective effect (beta coefficient = −1.46, SE: 0.82, *p*-value < 0.05) of the former DP in the main analysis and a detrimental effect (beta coefficient = 2.32, SE: 1.27, *p*-value < 0.05) of the latter DP for the more severe form of RA, respectively.

Separate models including single DPs provided estimates comparable to those from the composite model (data not shown). In the sensitivity analyses on participants who either had normal blood pressure or did not report gastro-esophageal reflux or gastritis, the point estimates were in line with those from the main analysis; however, the standard errors in robust linear regressions or the CIs in logistic regressions were sometimes wider, due to the smaller sample sizes in the stratified analyses. For example, for DAS-CRP, normal blood pressure subjects showed an OR of RA disease activity equal to 0.47 (95% CI: 0.22–1.00), as compared to the overall OR of 0.39 (95% CI: 0.21–0.74), for the highest versus the lowest category of the VUFA DP; similarly, subjects not reporting gastro-esophageal reflux or gastritis had an OR of 0.37 (95% CI: 0.18–0.79) for the same category of the VUFA DP. When we considered the SDAI disease outcome and the “Animal products” DP, the former group had an OR of 0.34 (95% CI: 0.14–0.85) and the latter group of 0.43 (95% CI: 0.18–1.01), as compared to the overall OR of 0.46 (95% CI: 0.23–0.94) for the intermediate consumption category in the main analysis.

## 4. Discussion

In this Italian cross-sectional study on RA disease activity, we identified five major DPs that explained ~80% of the nutritional variability in this population. Among the retained DPs, higher consumptions of the VUFA, AUFA, and possibly the “Animal products” DPs were related with a decreased risk of disease activity in logistic models or with a decreased mean disease activity in linear models, after mutual adjustment for all the remaining DPs and major confounders. The results were generally stronger in the stratified analyses for RA severity or duration, although the sample sizes were smaller than in the main analysis. In the highest category of consumption, the VUFA DP exerted significant protections against development of disease activity that ranged from ~60 to 90% across all the analyses and disease activity outcomes; similarly, the significant protections provided by the highest consumption of the AUFA DP were ~30%. In robust linear regressions, the decrease in DAS28-CRP ranged from 0.36 to 0.81 for the VUFA and from 0.25 to 0.71 for the AUFA DP; the decrease in SDAI ranged from 2.78 to 4.60 for the VUFA and from 3.85 to 4.75 for the AUFA DP. Finally, intermediate categories of consumption of the “Animal products” DP were related to the SDAI only: significant risk reductions ranged from ~0.55 to ~80% and significant decreases in SDAI ranged from 1.51 to 3.01.

The observed decreases in DAS28-CRP and SDAI are of clinically important magnitude. A similar publication [15] on consumption of fish—as a whole food—among 176 RA patients from a cross-sectional US study (DAS28-CRP: median: 3.5, IQR: 2.9–4.3) showed a statistically significant difference of 0.49 between the highest and lowest categories of consumption. This result is in line with those from our sample, where, however, we were able to reach 0.70 and 0.80 with a likely less compromised population (DAS28-CRP: median: 2.21, IQR: 1.61–3.02). We also agree with the authors [15] who concluded in favor of the clinical significance of their result: 0.49 was one third the magnitude of pre- and post-treatment DAS28 differences (e.g., mean decrease: 1.6) among methotrexate users from a trial including RA patients with moderate-to-high disease activity [52]. Following the same argument, we could compare our results on DAS28-CRP and SDAI with thresholds of minimal clinically important improvements in clinical trials [53]. In a population of RA patients with active disease following treatment escalation, minimal clinically important improvements with specificity equal to 0.80 were equal to −1.02 (CI: −1.44–−0.83) for DAS28-CRP and −13.1 (CI: −17.5–−10.7) for SDAI [54]. In our worst scenario, the DAS28-CRP reduction of 0.36 is approximately one third the magnitude of 1.02, but, when it reaches 0.81, it is at the 80% of the minimal clinically important improvement [54]; our SDAI decrease of 4.60 is still more than one third the required improvement of 13 [54]. In addition, in the study sample [54], the mean (±standard deviation) of DAS28-CRP was 5.55 ± 1.09 vs. 2.46 ± 1.12 in our sample, and the mean of SDAI 38.6 ± 14.8 vs. 9.01 ± 9.16 in our sample, thus pointing out that minimal clinically important improvements were calculated on a population with a definitely higher disease activity than ours.

Correlation coefficients between the identified DPs and selected food groups confirmed the labeling of DPs and provided further suggestions for comparing our results with those available in previous literature.

The VUFA DP was mainly characterized by consumption of nuts, olives, and olive oil, typically included in the Mediterranean diet. In the first case-control study on RA ever [55], a higher consumption of olive oil in Greece did not significantly correlate with RA duration or severity (according to the Ritchie index), although an increase in consumption by two times per week resulted in a significant relative risk of RA of 0.49 in multiple regression models adjusted also for fish and adherence to the Orthodox lent. Olive oil—as a single component of the Mediterranean diet—was not investigated in its effect on RA disease activity in the previous analysis based on a subset of the current study [20]. In addition, a case-control study based on the baseline data of the “TOtal Management Of Risk factors in Rheumatoid arthritis patients to lOWer morbidity and mortality” (TOMORROW) cohort study [25] assessed the role of the Mediterranean diet in RA development and disease activity in a population of Japanese participants, mainly composed by elderly women. Among single components of the Mediterranean diet, the ratio of monounsaturated fatty acids to saturated fatty acids significantly and negatively correlated with DAS28-ESR at baseline, after adjustment for subject’s age [25]. In addition, in this population (DAS28-ESR at baseline median: 3.44, IQR: 2.44–4.30), this ratio was significantly lower in subgroups with moderate and high disease activity (as compared to remission and low disease activity); a higher monounsaturated fatty acids intake was also associated with remission with borderline significance (OR = 1.97, 95% CI: 0.98–3.98), in a multiple regression model [25]. The protective role of high monounsaturated fatty acids—in absolute value or relative to saturated fatty acids—found in the TOMORROW study [25] is confirmed by our results on the VUFA DP, where monounsaturated fatty acids loads 0.62 and are close to be dominant nutrients for this DP. Unfortunately, we cannot have additional information on linoleic and linolenic fatty acids or vitamin E from the TOMORROW study which directly targeted Mediterranean diet and its components. A few clinical trials have considered supplementation with oils of vegetable source, including supplementation with evening primrose oil integrated with alpha-tocopherol (vs. olive oil) [12] and supplementation with vitamin E [56], with nongeneralizable [12] or null results [56]. Besides the well-known antioxidant properties of medium-chain fatty acids and vitamin E, their anti-inflammatory effects develop in reducing the expression of pro-inflammatory cytokines involved in the progression of RA, such as Interleukin (IL)-1Beta, IL-17, and Tumor Necrosing Factor (TNF)-alpha [8]; in addition, it is posited that the down-regulation of the expression of vascular cell adhesion molecules and intracellular adhesion molecules on immune system cells and vascular endothelial cells modulates the inflammatory response in RA [6].

We observed that the AUFA DP exerts a beneficial effect on disease activity. This DP reflects consumption of fish in general and especially of fatty fish, including (fresh or canned) tuna and (fresh or smoked) salmon in the current data collection. Red and white meat were also represented as minor contributors to this DP (i.e., corresponding correlation coefficients did not reach the cut-off of 0.30), thus reflecting the modest loading of arachidonic acid, which was, however, the highest loading across all DPs. The high loading of vitamin D on the AUFA DP, as well as EPA and DHA, suggested an animal source of fatty acids characterizing this DP, with a major role of fish and fatty fish. Our effect estimates were similar in magnitude to the mentioned US-based cross-sectional study [15] on consumption of fat nonfried fish of 176 RA patients, who showed, however, a more severe disease activity at baseline than our sample. Similarly, we found that fat (nonfried) fish shows the highest correlation coefficient (0.70) with the AUFA DP, thus confirming their hypothesis [15] that if the beneficial effect should come from the omega-3 fatty acid content of EPA + DHA, it is important to target nonfried fatty fish and to exclude other seafood items (e.g., fried fish, nonfried shellfish, and fish in mixed dishes) from the analysis. Our lean fish food group was still highly correlated with the AUFA DP because it likely contains also cod, mackerel, anchovies, and soles. However, our FFQ does not allow to assess if these fish items were consumed fried or not. The Greek study by Linos and coauthors [55] may be also used to investigate on a possible relationship between fish consumption and RA disease activity, but it did not provide a clear indication that longer or more severe RA variants correlated with a higher fish consumption. In the Japanese TOMORROW study [25], seafood consumption—as a component of the Mediterranean diet—was not materially different among RA patients subclassified according to severity of disease activity using DAS-ESR. Finally, no information on the effect of single components of the Mediterranean diet on RA disease activity was provided in the previous analysis based on a subset of the current study [20]. Although still weak, evidence from our and the previously mentioned observational studies agrees with long-standing evidence from randomized controlled trials on the effect of fish oil supplementation in RA [6,57]. Based upon a strong scientific rationale [58,59,60], the literature currently supports a beneficial effect of moderate-to-high doses of omega-3 fatty acids (e.g., ~3 up to 9 g EPA and DHA per day) on several outcomes of disease activity. Among possible mechanisms, we mention the decrease in inflammatory eicosanoids—lipid mediators of inflammation—the inhibition of the nod-like receptor (NLRP3) inflammasome, and the dampening in the production of inflammatory cytokines and chemokines, which leads to the resolution of inflammation through specialized pro-resolving mediators [6]. In addition, fish oil shows advantages in reducing cardiovascular events via nonsteroidal anti-inflammatory drug-sparing [60]; more recently, the benefit-risk balance of omega-3 acid ethyl esters medicinal products for oral use in secondary prevention after myocardial infarction was reconsidered and evaluated to be not favorable by the European Medicines Agency [61].

We observed a statistically significant protective effect on SDAI of intermediate scores of the “Animal products” DP, which was mainly characterized by consumption of cheese, milk, and yogurt, with red meat, butter/margarine, and sweets (mainly bakery products, jam, chocolate, and ice cream) being also present in the identified DP. We were unable to find out recent scientific literature on the role of meat and dairy products on disease activity in humans. We can hypothesize this mixed result is related to allergies and sensitivity to dairy products. Some of the patients likely experienced a detrimental effect of this DP due to sensitivity to dairy products, as suggested in an old systematic review citing a controlled study where after fasting or on a severely restricted diet, patients had a temporary but significant improvement in the signs and symptoms of RA; the improvement disappeared when milk was reintroduced into the diet [62]. The remaining part of the sample likely experienced a statistically significant protective effect of dairy products and meat, which was attenuated by the subgroup of patients showing allergies. Analyses on the consumption of milk and dairy products as risk factors for RA development yielded inconclusive results too (see [63] for recent null results based on the Swedish Mammography Cohort Study and [64] for a recent summary of the evidence on the effects of milk).

We observed a statistically significant detrimental effect of scoring high on the “Anti-oxidant vitamins and fiber” DP—correlating high with fruit and vegetables—on both DAS28-CRP and SDAI in the stratum of RF and/or ACPA positive subjects in robust linear models. The statistical significance was modest (*p*-value < 0.05) and the identified increases were not clinically relevant (~0.35 for DAS28-CRP and 2.32 for SDAI). A possible explanation is based on the key role of soluble carbohydrates. Our “Anti-oxidant vitamins and fiber” DP is more oriented toward fruit than vegetables. Soluble carbohydrates derived from fresh and processed fruit are likely to have a detrimental effect on RA disease activity. Likewise, De Christopher [65] discussed the role of ingested fructose leading to the formation of advanced glycation end products that travel beyond the intestinal boundaries to other tissues and may play a role in the etiology of auto-immune arthritis [64,66]. To integrate our results, higher intakes of fruit were also found to decrease the OR of remission in the TOMORROW study (OR = 0.49, 95% CI: 0.47–0.92), thus suggesting a trend similar to our study in their main analysis [25]. In general, laboratory studies and early trials suggested that a DP based on fruit should exert a beneficial effect on RA disease activity [10], in consideration of the well-known role of antioxidants in protecting against tissue damage caused by oxygen free radicals [57]. However, most of the current evidence is still based on trials proposing supplementation with uncommon dietary compounds [e.g., pomegranate extract [67], quercetin [68], cranberry juice [69], or ethnic fruit (e.g., Russian olives and figs [70])] and do not include commonly consumed fruit (e.g., berries), in doses resembling everyday life [10]. In addition, nightshade vegetables (eggplants, peppers, and tomatoes)—included in the “other vegetables” food group correlating high with our “Anti-oxidant and vitamins fiber” DP—are often mentioned by patients and discussed in patient-oriented websites as exacerbating RA symptoms. However, until today, published, peer-reviewed literature has never addressed the role of nightshade vegetables in RA disease activity [6].

Taken together, the five identified DPs disentangle the different dimensions of a Mediterranean dietary profile, including an abundance of plant-based foods, such as unrefined grains, fruit, vegetables, legumes, and olive oil, a moderate consumption of poultry, dairy products, and eggs, and a low consumption of red meat and sweets. When entered together in the same regression models, each of the retained DPs is related to RA activity, after controlling for the effect of all the remaining DPs. These two aspects are of great importance and justify the application of the a posteriori approach, as compared to the a priori one, in the current set-up.

Indeed, in the two observational studies considering the effect of the (a priori) Mediterranean DP on disease activity [20,25]—one based on a subset of the current database [3]—the total score was not significantly related to disease activity; however, when separately analyzed, single components of the Mediterranean diet exerted some beneficial effect [25]. Similarly, a weak evidence derived from the few trials on Mediterranean diet and disease activity outcomes, including also the DAS28 [16,21]. Benefits in the Mediterranean diet group were either modest and not significant for the DAS28 [21] or, when present for DAS28, difficult to extend to other populations (i.e., the Mediterranean diet was adapted to suit Swedish subjects) [16]. These results seem unexpected based on the biological arguments previously provided for the single Mediterranean diet components and for the important role that microbiome-derived dietary metabolites (e.g., short-chain, medium-chain, and omega-3 long-chain fatty acids) have on the immune cell function through G-protein-coupled receptors (GPR) [6]. One possible explanation for this apparent contradiction is that the single Mediterranean diet components are related to disease activity in opposite ways. If this is the case, separating out the role of subsets of food groups or nutrients with the a posteriori DPs is a promising strategy.

In addition, the adjustment of each DP for the remaining DPs provides a clearer picture of the effective contribution of each DP and of the related nutrients/food groups. For example, the protection provided by VUFA DP—correlating high with nuts and olive oil intakes—was found after the adjustment for the “Anti-oxidant vitamins and fiber” DP—correlating high with fruit and vegetables; this allows concluding that dressing itself was the major contributor to disease activity, whereas raw or cooked vegetables are likely to have a minimal role, if any. Similarly, the protection provided by fatty and lean fish in the AUFA DP was found after the adjustment for the VUFA DP, thus suggesting an independent role of unsaturated fats from either source. Finally, when the total variance explained by the retained DPs is high (~80% in our analysis), entering the five DPs simultaneously in the same regression model provides a valid alternative to the usual adjustment for total energy intake, suggested in any analysis of a priori DPs.

The current study has strengths and limitations. Among the strengths, we expanded the list of nutrients used in previous papers of our group on several cancer sites (e.g., [43,44,45]) to include nutrients such as EPA, DHA, lignans, flavonoids, phenolic acids, and tyrosols, which are likely to be involved in RA development or disease activity [6,7,8,9,13,14]. Other nutrients could be added to our list. For example, differences within saturated and monounsaturared fatty acids could be done because some of them, including palmitic and stearic acids within the saturated or palmitoleic and oleic acids within the monosaturated fatty acids, have different potential biological activities to be investigated. However, the number of nutrients included in our list was comparable to—or even larger than—most of the papers on a posteriori DPs [17,19]; these nutrients represented well the overall diet, with a good balance between its different components. For example, if we had to add detailed information on single saturated and monounsaturared fatty acids, the fat profile would be overrepresented. Moreover, our FFQ [34] collected major food sources of the nutrients selected for the current analysis [36,37,39,49]. In addition, we are aware of the importance of confounding factors in the relationship between diet and RA disease activity [6]. We adjusted our regression models for several sociodemographic, anthropometric, and lifestyle factors, as well as medications, and clinical course information, thus reducing possible residual confounding. However, many unmeasured or difficult-to-measure potential confounders still exist (e.g., depression and sleep quantity and quality) and this may compromise the validity of our results. Among the study limitations, we mention its cross-sectional design, which does not allow to draw firm conclusions about the impact of DPs on RA disease activity. Reverse causation is still a possible explanation for with observed a relation between the identified DPs and disease activity; prospective studies are suggested to minimize this potential source of bias. We plan to further explore the role of DPs on disease activity referring to the follow-up wave of the current study, where information from the same FFQ, laboratory parameters, therapy, and disease activity was recently collected. In addition, although the study has a reasonable sample size, obtaining precise estimates in stratified analyses would have likely required an even larger database. Further issues are related to the use of factor analysis in the identification of a posteriori dietary patterns [19]. This technique requires subjective decisions at various stages of the analysis, including the type and number of dietary components to include, the number of factors to retain, the choice of applying a rotation method or not (and which method to use), and the labeling and interpretation of the retained factors. As a few studies have assessed the effects of these decisions on the final factor analysis solution [71], we inspected results from several complementary analyses and referred to different available criteria for any step in our decision process. The results were not materially different at any stage and this reassures that the identified DPs do exist in this Italian population. In addition, the reproducibility of the a posteriori DPs across different studies, populations, countries and over time is still a matter of concern [72]. Given that this is the first study that derived a posteriori DPs in a RA population, we hope additional studies will be published soon to compare results with ours.

## 5. Conclusions

To our knowledge, this paper is the first attempt to provide a comprehensive description of actual dietary habits in RA subjects with the identification of a posteriori DPs. In line with the available evidence on DPs and RA disease activity, both an animal and a vegetable unsaturated fatty, acids DPs were identified, as well as other DPs typical of the Italian tradition [73], such as the “Starch-rich” and the “Animal products” DPs. The additional modeling effort required by a posteriori DPs has ended up in statistically and clinically significant reductions in RA disease activity of the DPs based on vegetable or animal unsaturated fatty acids, especially when more severe or long-standing RA variants were considered. In addition, the protection provided by vegetable oils was found after the adjustment for a DP high in fruit and vegetables, thus pointing to a major role of dressing, as compared to raw or cooked vegetables. Similarly, the protection provided by fish was identified after the adjustment for vegetable oils, thus suggesting an independent role of unsaturated fatty acids from either source.

## Figures and Tables

**Table 1:** Factor loading matrix ^1^, communalities (COMM) and explained variances (VAR) for the five major dietary patterns identified by principal component factor analysis.

Nutrient	Dietary Patterns					COMM
	Anti-Oxidant Vitamins and Fiber	Starch-Rich	VUFA ^2^	AUFA ^2^	Animal Products	
Total protein	−0.39	**0.74**	−0.19	0.25	−0.38	0.95
Cholesterol	−0.21	0.35	-	0.32	**−0.80**	0.90
Saturated fatty acids	−0.22	0.37	−0.21	-	**−0.83**	0.92
Monounsaturated fatty acids	−0.17	0.41	−0.62	0.11	−0.56	0.90
Linoleic acid	-	0.31	**−0.87**	-	−0.21	0.91
Linolenic acid	−0.18	0.16	**−0.85**	-	−0.18	0.82
Arachidonic acid	−0.13	0.23	-	0.47	−0.37	0.42
EPA ^2^	−0.14	-	−0.11	**0.95**	-	0.94
DHA ^2^	−0.13	-	−0.11	**0.95**	-	0.95
Soluble carbohydrates	**−0.78**	0.27	−0.22	-	−0.26	0.81
Starch	-	**0.81**	-	-	−0.14	0.69
Sodium	-	**0.69**	−0.15	-	−0.36	0.63
Calcium	−0.56	0.41	−0.13	-	−0.57	0.83
Potassium	**−0.80**	0.40	−0.30	0.20	−0.18	0.96
Phosphorus	−0.49	**0.66**	−0.26	0.18	−0.38	0.93
Iron	−0.59	**0.65**	−0.31	0.19	−0.12	0.92
Zinc	−0.49	**0.64**	−0.25	0.18	−0.28	0.83
Magnesium	−0.57	**0.68**	−0.36	0.13	-	0.93
Copper	−0.53	0.61	−0.49	0.23	-	0.95
Selenium	−0.14	**0.89**	-	0.12	−0.14	0.85
Thiamin (vitamin B1)	−0.46	**0.67**	−0.30	0.15	−0.17	0.80
Riboflavin (vitamin B2)	−0.56	0.53	−0.21	0.10	−0.36	0.78
Niacin	−0.42	**0.74**	−0.20	0.34	−0.12	0.89
Vitamin C	**−0.92**	-	−0.15	0.15	-	0.91
Vitamin A—RAE	**−0.81**	0.15	−0.15	0.14	−0.32	0.81
Vitamin D	−0.13	0.10	−0.10	**0.93**	−0.10	0.92
Vitamin E	−0.50	0.29	**−0.65**	0.12	−0.19	0.80
Soluble Fiber	**−0.78**	0.36	−0.17	-	-	0.78
Insoluble Fiber	**−0.76**	0.39	−0.24	0.15	-	0.82
Lignans	**−0.79**	-	-	-	−0.11	0.65
Flavonoids	**−0.68**	-	−0.21	0.15	-	0.53
Phenolic acids	−0.27	-	−0.44	-	0.21	0.31
Tyrosols	−0.14	-	−0.37	0.26	-	0.23
Proportion of explained VAR (%)	25.05	22.12	11.76	11.07	9.85	
Cumulative explained VAR (%)	25.05	47.17	58.93	70.00	79.85	

^1^ Estimates from a principal component factor analysis carried out on 33 nutrients. The greater (in absolute value) was the loading of a given nutrient to a factor, the higher was the contribution of that nutrient to the factor. For each factor, loadings greater or equal to 0.63 (in absolute value) were meant to indicate important or “dominant nutrients” and were shown in bold typeface; loadings smaller than 0.1 in absolute value were suppressed. ^2^ AUFA: Animal unsaturated fatty acids; DHA: Docosahexaenoic acid; EPA: Eicosapentaenoic acid; RAE: Retinol activity equivalent; VUFA: Vegetable unsaturated fatty acids.

**Table 2:** Spearman rank correlation coefficients ^1^ between nutrient-based dietary patterns (factor scores) derived from principal component factor analysis and daily frequencies of consumption for selected food groups and condiments derived on the same data.

Food Group	Anti-Oxidant Vitamins and Fiber	Starch-Rich	VUFA ^2^	AUFA ^2^	Animal Products
Red meat	-	0.18	-	0.20	**0.38**
White meat	-	0.10	-	0.16	0.20
Lean fish	-	-	0.11	**0.60**	-
Fatty fish	-	-	0.14	**0.70**	-
Seafood	-	-	-	0.29	-
Eggs	0.11	0.12	0.11	-	0.14
Sweets	-	0.23	0.15	-0.11	**0.34**
Snacks	-	0.17	0.18	-	0.22
Dried fruit	0.14	0.18	0.19	0.13	-
Nuts	0.11	0.11	**0.76**	0.15	-
Olive oil and olives	0.14	0.12	**0.49**	0.17	-
Seed oil	-	-	0.18	-	-
Leafy vegetables	**0.45**	0.15	0.21	0.15	-
Cabbages	**0.33**	0.15	0.21	0.18	-
Legumes	**0.30**	0.29	0.28	0.24	-
Onion and garlic	0.20	0.19	0.11	0.18	-
Mushrooms	0.10	-	0.15	0.14	0.15
Other Vegetables	**0.53**	0.21	0.29	0.15	-
Soy products	-	-	0.19	0.10	-
Fruit, all seasons	**0.66**	0.14	0.18	-	-
Fruit, winter season	**0.72**	-	-	-	0.13
Fruit, summer season	**0.67**	0.11	0.23	-	0.10
Tea and herbal tea	0.25	-	0.18	0.13	-
Coffee	-	0.22	-	-	-
Alcoholic beverages	-	-	0.14	-	-
Soft drinks	-	-	-	-	0.23
Refined grains	-	**0.76**	-	−0.13	0.14
Potatoes	-	**0.30**	0.16	-	0.25
Whole grains	0.24	**0.40**	0.14	-	-
Butter and margarine	-	-	-	-	**0.34**
Milk and yogurt	0.16	0.23	-	-	**0.37**
Hard cheese	0.20	0.10	-	-	**0.61**
Soft cheese	0.19	0.23	-	-	**0.45**

^1^ Correlations greater or equal to 0.30 (in absolute value) were shown in bold typeface; correlations smaller than 0.1 (in absolute value) were suppressed. ^2^ AUFA: Animal unsaturated fatty acids; VUFA: Vegetable unsaturated fatty acids.

**Table 3:** Odds Ratios (ORs) of rheumatoid arthritis disease activity and corresponding 95% confidence Intervals (CIs) (upper panel) and increments in the mean DAS-CRP in continuous (lower panel), according to the highest tertile-based categories of consumption of five retained dietary patterns from a principal component factor analysis ^1,2^.

		**Overall Analysis**	**FR and/or ACPA Positivity**		**Disease Duration > 15 Years**	
**Logistic Regression**						
	**Tertile**	**OR (95% CI)**	**OR (95% CI)**	**p_hetero_^3^**	**OR (95% CI)**	**p_hetero_^3^**
Anti-oxidant vitamins and fiber	Q1–Q2	1.52 (0.81–2.84)	2.59 (1.00–6.70)		0.92 (0.30–2.85)	
	≤Q1	1.74 (0.91–3.31)	2.21 (0.86–5.69)	0.50	1.05 (0.34–3.27)	1.00
Starch-rich	Q1–Q2	0.76 (0.40–1.42)	0.72 (0.30–1.72)		0.45 (0.14–1.47)	
	>Q2	0.82 (0.44–1.56)	1.08 (0.44–2.67)	0.70	0.54 (0.19–1.56)	0.21
VUFA ^4^	Q1–Q2	0.51 (0.27–0.95)	0.39 (0.16–0.92)		0.18 (0.05–0.58)	
	≤Q1	0.39 (0.21–0.74)	0.18 (0.07–0.46)	0.13	0.10 (0.03–0.36)	0.11
AUFA ^4^	Q1–Q2	0.73 (0.39–1.38)	0.53 (0.22–1.30)		0.67 (0.21–2.12)	
	>Q2	0.53 (0.28–1.00)	0.33 (0.13–0.82)	0.16	0.29 (0.09–0.92)	0.69
Animal products	Q1–Q2	0.80 (0.42–1.53)	0.96 (0.39–2.37)		0.68 (0.23–2.08)	
	≤Q1	0.81 (0.44–1.50)	0.73 (0.31–1.71)	0.93	1.17 (0.40–3.43)	0.39
**Robust Linear Regression** **^5^**						
	**Tertile**	**Beta (SE)**	**Beta (SE)**	**p_hetero_** **^3^**	**Beta (SE)**	**p_hetero_** **^3^**
Anti-oxidant vitamins and fiber	Q1–Q2	013 (0.14)	0.34 (0.20) *		0.05 (0.25)	
	≤Q1	0.22 (0.14)	0.37 (0.20) *	0.42	0.02 (0.25)	1.00
Starch-rich	Q1–Q2	−0.12 (0.14)	−0.05 (0.19)		0.03 (0.25)	
	>Q2	−0.09 (0.14)	0.01 (0.20)	0.87	−0.09 (0.23)	0.81
VUFA ^4^	Q1–Q2	−0.32 (0.14) **	−0.36 (0.19) *		−0.65 (0.24) ***	
	≤Q1	−0.36 (0.14) ***	−0.53 (0.19) ***	0.78	−0.81 (0.25) ***	0.22
AUFA ^4^	Q1–Q2	−0.25 (0.14) *	−0.56 (0.19) ***		−0.54 (0.25) **	
	>Q2	−0.25 (0.14) *	−0.53 (0.20) ***	0.11	−0.71 (0.24) ***	0.25
Animal products	Q1–Q2	−0.13 (0.14)	0.06 (0.20)		−0.22 (0.25)	
	≤Q1	−0.07 (0.14)	−0.12 (0.19)	0.38	0.00 (0.24)	0.83

^1^ Estimates from unconditional logistic or robust linear regression models adjusted for age, sex, education, body mass index, cigarette smoking status, alcohol drinking intensity, disease duration, rheumatoid factor, anti-citrullinated protein antibodies, presence of any therapy, csDMARDS, bDMARDS, tsMARDS, and steroids, when possible. The results refer to the composite model including all the five factors simultaneously. ^2^ The reference category included the lowest consumers of each dietary pattern. This corresponded to the lowest tertile category for patterns characterized by positive factor loadings, and the highest tertile category for those patterns characterized by negative factors loadings. ^3^ *p*-value for heterogeneity of effects estimates across strata. ^4^ AUFA: Animal unsaturated fatty acids; VUFA: Vegetable unsaturated fatty acids. ^5^ *p*-value from a Student *t*-test on single beta coefficients. Significance was indicated as follows: 0 ‘***’ 0.001 ‘**’ 0.01 ‘*’ 0.05 ‘.’ 0.1 ‘ ’ 1.

**Table 4:** Odds Ratios (ORs) of rheumatoid arthritis disease activity and corresponding 95% confidence Intervals (CIs) (upper panel) and increments in the mean SDAI in continuous (lower panel), according to the highest tertile-based categories of consumption of five retained dietary patterns from a principal component factor analysis ^1,2^.

		**Overall Analysis**	**FR and/or ACPA Positivity**		**Disease Duration > 15 Years**	
**Logistic Regression**						
	**Tertile**	**OR (95% CI)**	**OR (95% CI)**	**p_hetero_^3^**	**OR (95% CI)**	**p_hetero_^3^**
Anti-oxidant vitamins and fiber	Q1–Q2	0.77 (0.39–1.50)	0.91 (0.36−2.31)		0.63 (0.17−2.34)	
	≤Q1	1.24 (0.62−2.49)	1.52 (0.57−4.01)	0.89	1.16 (0.30−4.58)	0.61
Starch-rich	Q1–Q2	0.70 (0.35−1.39)	0.75 (0.30−1.84)		0.63 (0.17−2.37)	
	>Q2	0.70 (0.35–1.41)	0.83 (0.32−2.19)	0.75	0.79 (0.23−2.70)	0.87
VUFA ^4^	Q1–Q2	0.75 (0.37−1.52)	0.44 (0.16−1.18)		0.79 (0.20−3.13)	
	≤Q1	0.71 (0.36−1.40)	0.36 (0.14−0.93)	0.11	0.57 (0.15−2.23)	0.80
AUFA ^4^	Q1–Q2	0.76 (0.39−1.48)	0.52 (0.21−1.30)		0.27 (0.07−1.02)	
	>Q2	0.82 (0.41−1.66)	0.78 (0.29−2.15)	0.32	0.44 (0.12−1.61)	0.17
Animal products	Q1–Q2	0.46 (0.23−0.94)	0.87 (0.33−2.32)		0.22 (0.05−1.00)	
	≤Q1	0.53 (0.27−1.04)	0.82 (0.33−2.08)	0.16	0.34 (0.09−1.32)	0.82
**Robust Linear Regression ^5^**						
	**Tertile**	**Beta (SE)**	**Beta (SE)**	**p_hetero_^3^**	**Beta (SE)**	**p_hetero_^3^**
Anti-oxidant vitamins and fiber	Q1–Q2	0.92 (0.81)	2.32 (1.27) *		0.81 (1.62)	
	≤Q1	0.94 (0.82)	1.91 (1.26)	0	-0.18 (1.64)	1
Starch-rich	Q1–Q2	−1.46 (0.82) *	−0.26 (1.21)		0.22 (1.68)	
	>Q2	−0.88 (0.83)	−0.61 (1.26)	1	−0.96 (1.53)	0
VUFA ^4^	Q1–Q2	−0.65 (0.81)	−1.72 (1.22)		−3.53 (1.59) **	
	≤Q1	−1.27 (0.80)	−2.78 (1.22) **	1	−4.60 (1.66) ***	0
AUFA ^4^	Q1–Q2	−1.01 (0.81)	−3.56 (1.22) ***		−3.09 (1.68) *	
	>Q2	−1.08 (0.83)	−3.85 (1.27) ***	0	−4.75 (1.61) ***	0
Animal products	Q1–Q2	−1.51 (0.83) *	−0.65 (1.26)		−3.01 (1.63) *	
	≤Q1	−1.05 (0.80)	−1.30 (1.22)	0	−0.77 (1.59)	0

^1^ Estimates from unconditional logistic or robust linear regression models adjusted for age, sex, education, body mass index, cigarette smoking status, alcohol drinking intensity, disease duration, rheumatoid factor, anti-citrullinated protein antibodies, presence of any therapy, csDMARDS, bDMARDS, tsMARDS, and steroids, when possible. The results refer to the composite model including all the five factors simultaneously. ^2^ The reference category included the lowest consumers of each dietary pattern. This corresponded to the lowest tertile category for patterns characterized by positive factor loadings, and the highest tertile category for those patterns characterized by negative factors loadings. ^3^ *p*-value for heterogeneity of effects estimates across strata. ^4^ AUFA: Animal unsaturated fatty acids; VUFA: Vegetable unsaturated fatty acids. ^5^ *p*-value from a Student *t*-test on single beta coefficients. Significance was indicated as follows: 0 ‘***’ 0.001 ‘**’ 0.01 ‘*’ 0.05 ‘.’ 0.1 ‘ ’ 1.

## Supplementary Materials

The following are available online at https://www.mdpi.com/2072-6643/12/12/3856/s1, Table S1: Factorability of the nutrient-based correlation matrix: Bartlett’s test of sphericity and measures of sampling adequacy, Table S2: Factorability of the nutrient-based correlation matrix: Bartlett’s test of sphericity and measures of sampling adequacy, Table S3: Odds Ratios (ORs) of rheumatoid arthritis disease activity and corresponding 95% confidence Intervals (CIs) (upper panel) and increments in the mean Disease Activity Score on 28 joints with C-reactive protein in continuous (lower panel), according to the highest tertile-based categories of consumption of five retained dietary patterns from a principal component factor analysis ^1,2,3^. Results were presented in strata of less severe rheumatoid arthritis and shorter disease durations, Table S4: Odds Ratios (ORs) of rheumatoid arthritis disease activity and corresponding 95% confidence Intervals (CIs) (upper panel) and increments in the mean Simplified Disease Activity Index in continuous (lower panel), according to the highest tertile-based categories of consumption of five retained dietary patterns from a principal component factor analysis ^1,2,3^. Results were presented in strata of less severe rheumatoid arthritis and shorter disease durations.
