# Supplementary material for: A Posteriori Dietary Patterns and Rheumatoid Arthritis Disease Activity: A Beneficial Role of Vegetable and Animal Unsaturated Fatty Acids
Source: Nutrients. 2020 Dec 17;12(12):3856. doi: 10.3390/nu12123856 (PMC7766886; doi:10.3390/nu12123856)
Supplement: Supplementary file 1 [file nutrients-12-03856-s001.zip › Supplementary_material/SupplementaryTable4.docx]

**Table S4.** Odds Ratios (ORs) of rheumatoid arthritis disease activity and corresponding 95% confidence Intervals (CIs) (upper panel) and increments in the mean Simplified Disease Activity Index in continuous (lower panel), according to the highest tertile-based categories of consumption of five retained dietary patterns from a principal component factor analysis^1,2,3^. Results were presented in strata of less severe rheumatoid arthritis and shorter disease durations.

|  |  | RF and ACPA negative^4^ | Disease duration < 15 years |
| --- | --- | --- | --- |
| Logistic regression | | | |
|  | **Tertile** | **OR (**95% CI**)** | **OR (**95% CI**)** |
| Anti-oxidant vitamins and fiber | Q1-Q2 | 0.51 (0.14-1.82) | 0.87 (0.35-2.17) |
|  | ≤Q1 | 0.74 (0.19-2.82) | 0.96 (0.38-2.38) |
| Starch-rich | Q1-Q2 | 0.91 (0.24-3.51) | 0.78 (0.33-1.86) |
|  | >Q2 | 0.67 (0.18-2.52) | 0.91 (0.36-2.31) |
| VUFA^4^ | Q1-Q2 | 1.43 (0.39-5.27) | 0.48 (0.19-1.24) |
|  | ≤Q1 | 1.67 (0.41-6.82) | 0.52 (0.21-1.25) |
| AUFA^4^ | Q1-Q2 | 1.79 (0.49-6.52) | 1.20 (0.49-2.95) |
|  | >Q2 | 1.14 (0.33-3.98) | 0.85 (0.34-2.14) |
| Animal products | Q1-Q2 | 0.21 (0.05-0.85) | 0.46 (0.19-1.13) |
|  | ≤Q1 | 0.26 (0.07-0.91) | 0.58 (0.24-1.40) |
|  | | | |
| Robust linear regression^5^ | | | |
|  | Tertile | **Beta (SE)** | **Beta (SE)** |
| Anti-oxidant vitamins and fiber | Q1-Q2 | -0.69 (1.35) | 0.45 (1.07) |
|  | ≤Q1 | 0.21 (1.43) | 0.68 (1.07) |
| Starch-rich | Q1-Q2 | -1.71 (1.45) | -0.93 (1.04) |
|  | >Q2 | -2.09 (1.43) | -0.78 (1.09) |
| VUFA^4^ | Q1-Q2 | -0.86 (1.46) | -1.82 (1.07)* |
|  | ≤Q1 | -1.14 (1.58) | -1.29 (1.04) |
| AUFA^4^ | Q1-Q2 | -0.41 (1.42) | -1.27 (1.06) |
|  | >Q2 | -0.70 (1.42) | -0.62 (1.09) |
| Animal products | Q1-Q2 | -2.07 (1.43) | -0.82 (1.05) |
|  | ≤Q1 | -0.30 (1.37) | -0.55 (1.02) |

^1^ Estimates from unconditional logistic or robust linear regression models adjusted for age, sex, education, body mass index, cigarette smoking status, alcohol drinking intensity, disease duration, rheumatoid factor, anti-citrullinated protein antibodies, presence of any therapy, conventional synthetic, biologic, targeted synthetic disease modifying anti-rheumatic drugs, and steroids, when possible. Results refer to the composite model including all the five factors simultaneously.  ^2^The reference category included the lowest consumers of each dietary pattern. This corresponded to the lowest tertile category for patterns characterized by positive factor loadings, and the highest tertile category for those patterns characterized by negative factors loadings. ^3^ The corresponding p-values for heterogeneity of effects estimates across strata were reported in Table 4. ^4^ ACPA: anti-citrullinated protein antibodies; AUFA: animal unsaturated fatty acids; RF: rheumatoid factor; VUFA: Vegetable unsaturated fatty acids. ^5^ P-value from a Student t-test on single beta coefficients. Significance was indicated as follows: 0 ‘***’ 0.001 ‘**’ 0.01 ‘*’ 0.05 ‘.’ 0.1 ‘ ’ 1.
