# Supplementary material for: A Posteriori Dietary Patterns and Rheumatoid Arthritis Disease Activity: A Beneficial Role of Vegetable and Animal Unsaturated Fatty Acids
Source: Nutrients. 2020 Dec 17;12(12):3856. doi: 10.3390/nu12123856 (PMC7766886; doi:10.3390/nu12123856)
Supplement: Supplementary file 1 [file nutrients-12-03856-s001.zip › Supplementary_material/SupplementaryTable2.docx]

**Table S2.** Factorability of the nutrient-based correlation matrix: Bartlett’s test of sphericity and measures of sampling adequacy.

| **Bartlett’s test of sphericity:** p-value< 0.001 | |
| --- | --- |
| **Overall measure of sampling adequacy (Kaiser-Meyer-Olkin statistic)^1^:** 0.88 | |
| **Individual measures of sampling adequacy^1^:** | |
| 0.30-0.69 | phenolic acids |
| 0.70-0.79 | tyrosols, DHA^2^, starch, linolenic acid |
| 0.80-0.89 | saturated fatty acids, linoleic acid, vitamin D, vitamin E, monounsaturated fatty acids, riboflavin (vitamin B2), cholesterol, vitamin C, arachidonic acid, EPA^2^, potassium, copper, thiamin (vitamin B1), selenium, soluble fiber |
| ≥0.90 | total protein, insoluble fiber, lignans, calcium, phosphorus, magnesium, niacin, soluble carbohydrates, iron, vitamin A – RAE, sodium, flavonoids, zinc |

^1^ Overall and individual measures of sampling adequacy range between 0 and 1, with values > 0.60 indicating a satisfactory size. ^2^ DHA: Docosahexaenoic acid; EPA: Eicosapentaenoic acid; RAE: Retinol activity equivalent.
