# Supplementary material for: A Posteriori Dietary Patterns and Rheumatoid Arthritis Disease Activity: A Beneficial Role of Vegetable and Animal Unsaturated Fatty Acids
Source: Nutrients. 2020 Dec 17;12(12):3856. doi: 10.3390/nu12123856 (PMC7766886; doi:10.3390/nu12123856)
Supplement: Supplementary file 1 [file nutrients-12-03856-s001.zip › Supplementary_material/SupplementaryTable1.docx]

**Table S1.** Distribution of 365 rheumatoid arthritis patients according to selected characteristics. Italy 2018-2019.

| **Characteristics** |  |
| --- | --- |
| **Age at baseline**, years, median (IQR) | 58.46 (47.81-69.03) |
| ≤55 years, N (%) | 155 (42.47) |
| >55, N (%) | 210 (57.53) |
| **Female**, N (%) | 287 (78.63) |
| **Education**^1^ |  |
| Primary school, N (%) | 49 (13.42) |
| Middle school, N (%) | 79 (21.64) |
| High school, N (%) | 134 (36.71) |
| University, N (%) | 87 (23.84) |
| **Body Mass Index**, Kg/m^2^, median (IQR) | 23.63 (21.00-26.78) |
| <18.5, N (%) | 22 (6.03) |
| 18.5 - 25, N (%) | 207 (56.71) |
| 25 - 30, N (%) | 94 (25.75) |
| ≥30, N (%) | 42 (11.51) |
| **Cigarette smoking status**^1^ |  |
| Never, N (%) | 189 (51.78) |
| Former, N (%) | 118 (32.33) |
| Current, N (%) | 55 (15.07) |
| **Alcohol drinking intensity** |  |
| Never, N (%) | 106 (29.04) |
| <1 drink/day, N (%) | 194 (53.15) |
| 1 - 2 drinks/day, N (%) | 29 (7.95) |
| ≥2 drinks/day, N (%) | 36 (9.86) |
| **Disease duration**, years, median (IQR) | 12.81 (8.08-20.72) |
| ≤5, N (%) | 51 (13.97) |
| 5 - 10, N (%) | 84 (23.01) |
| 10 - 15, N (%) | 76 (20.82) |
| 15 - 25, N (%) | 89 (24.38) |
| >25, N (%) | 65 (17.81) |
| Positivity for **rheumatoid factor**, N (%) | 196 (53.70) |
| Positivity for **anti-citrullinated protein antibodies**, N (%) | 186 (50.96) |
| **DAS28-CRP**, median (IQR) | 2.21 (1.61-3.02) |
| Remission, N (%) | 227 (62.19) |
| Low activity, N (%) | 60 (16.44) |
| Moderate activity, N (%) | 64 (17.53) |
| High activity, N (%) | 14 (3.84) |
| **SDAI**, median (IQR) | 6.30 (3.01-11.81) |
| Remission, N (%) | 108 (29.59) |
| Low activity, N (%) | 155 (42.47) |
| Moderate activity, N (%) | 81 (22.19) |
| High activity, N (%) | 21 (5.75) |
| **Swollen joint count** (0-28), median (IQR) | 0 (0-1) |
| **Tender joint count** (0-28), median (IQR) | 0 (0-2) |
| **C-reactive protein**, mg/dL, median (IQR) | 2 (0.6-5.57) |
| **General Health** (0-100), median (IQR) | 70 (60-85) |
| **Physician's Global Assessment** (0-10), median (IQR) | 2 (0-4) |
| **Comorbidities** |  |
| Chronic renal failure, N (%) | 4 (1.1) |
| Arterial hypertension, N (%) | 122 (33.42) |
| Coronary artery disease, N (%) | 19 (5.21) |
| Diabetes mellitus, N (%) | 19 (5.21) |
| Gastro-esophageal reflux disease, N (%) | 70 (19.18) |
| Inflammatory bowel disease, N (%) | 2 (0.55) |
| Gastritis, N (%) | 32 (8.77) |
| Esophagitis, N (%) | 5 (1.37) |

^1^ The sum does not add to the total because of a few missing values in education (16 subjects, 4.38%) and cigarette smoking status (3 subjects, 0.82%).
